# Incorporating practitioner knowledge to test and improve a new conceptual framework for healthy urban design and planning

**DOI:** 10.1080/23748834.2020.1773035

**Published:** 2020-06-08

**Authors:** Helen Pineo, Gemma Moore, Isobel Braithwaite

**Affiliations:** a Institute for Environmental Design and Engineering, Bartlett School of Environment, Energy and Resources, University College London, London, UK; b UCL Institute for Health Informatics, University College London, London, UK

**Keywords:** Urban, health and wellbeing, design, planning, participatory research

## Abstract

There are increasing arguments for bridging diverse knowledges and co-producing new knowledge between researchers, professional communities and citizens to create health-promoting built environments. The new THRIVES Framework (Towards Healthy uRbanism: InclusiVe, Equitable, Sustainable) echoes the call that healthy urbanism processes should be participatory and this principle informed the development of the Framework itself, which involved several stages of informal and formal testing with stakeholders, through a process of action research and ‘extended peer review’. Formal feedback about the design of the preliminary Framework and its implementation in built environment practice was gathered through a participatory workshop with 26 built environment and public health professionals in January 2020. Participants were encouraged to share their knowledge, ask questions, critique and provide recommendations. Overall, participants were supportive of the conceptual messages of the THRIVES Framework and more critical of the visual design of the preliminary version. They also questioned whether further resources would be required to implement the Framework. This research created a forum for stakeholders, who may typically be outside the research process, to shape the development of a conceptual framework for healthy urbanism. Further research and collaboration will create resources to bridge the gap between this new conceptualisation and practice.

## Introduction

Despite significant research knowledge about how the built environment affects health, delivering health and wellbeing objectives through urban planning and design is still a relatively specialist area for many professionals (Pilkington *et al*. 2013, Public Health England 2019). British practitioners often see healthy placemaking as being in competition with other development objectives and too costly to implement (Design Council 2018) and similar challenges have been raised in other countries (Kent and Thompson 2019). Yet the growing prevalence of chronic diseases and increasing healthcare costs, alongside pressing and interlinked environmental challenges such as pollution, climate change and biodiversity loss, clearly show that healthy and sustainable places have a key role to play in helping us to solve health and environmental crises in tandem (Younger *et al*. 2008, Gatzweiler *et al*. 2017, Opoku 2019). Furthermore, there are strong connections between environmental health and issues of justice and equity (Agyeman 2013), necessitating a holistic conceptualisation of healthy urban design and planning. These complex and pervasive challenges, and the limitations of built environment policy, planning and design in addressing them to date, demonstrate the need to empower built environment professionals with the knowledge and skills they need to achieve healthy placemaking.

**Figure 1:**
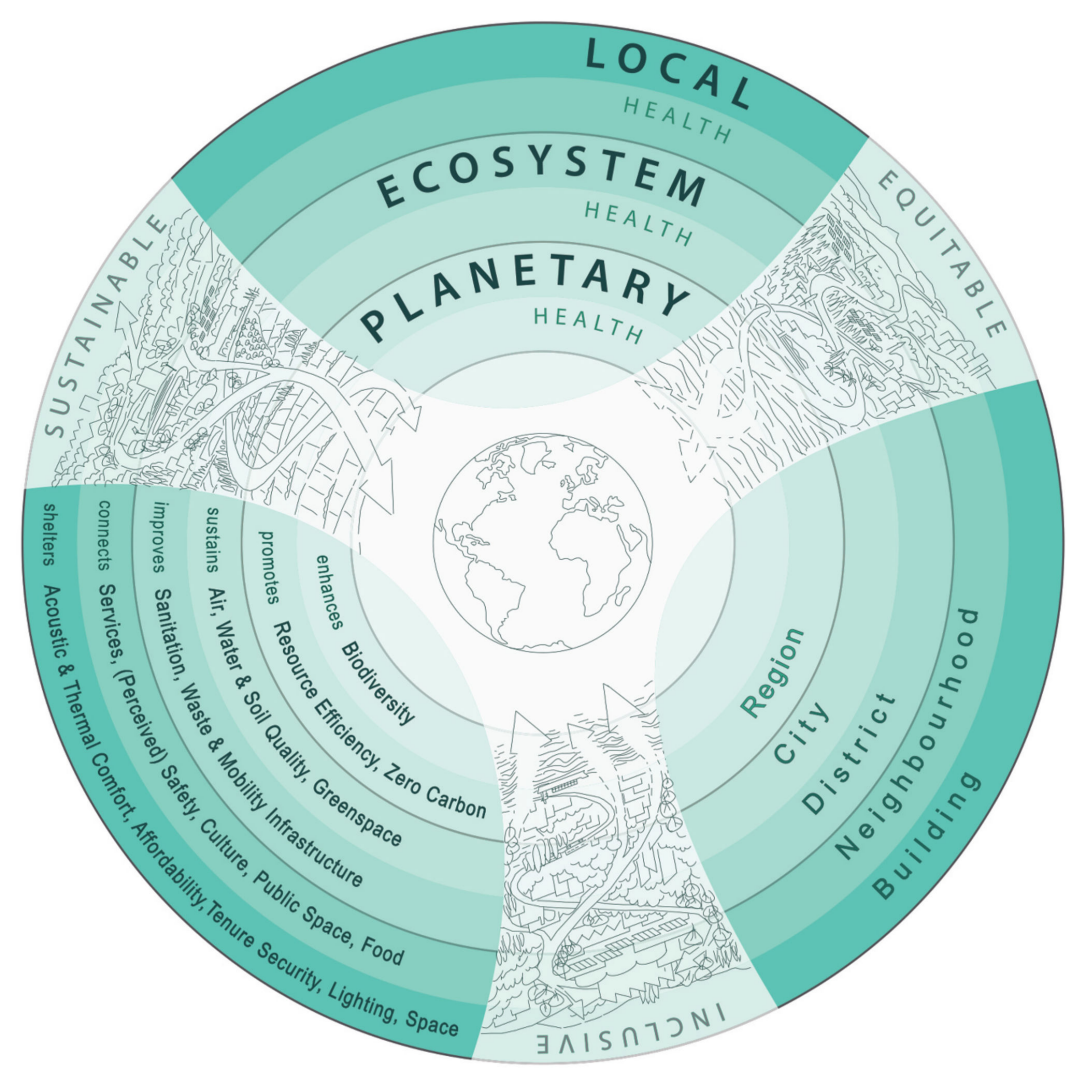
THRIVES framework (Towards Healthy Urbanism: InclusiVe Equitable Sustainable).

Existing frameworks for healthy urban design and planning address specific aspects of the built environment or determinants of health, but there is a need for a holistic conceptual framing that brings together impacts at diverse spatial and temporal scales and considers the interconnected goals of sustainability, equity and inclusion. Pineo (2020) builds upon and extends existing frameworks (e.g. Barton and Grant (2006) Health Map) to propose a new way of understanding the interconnected health impacts of policies and design decisions at multiple spatial and temporal scales − the THRIVES (Towards Healthy uRbanism: InclusiVe Equitable Sustainable) Framework (Figure 1). The Framework consists of three core principles − equity, sustainability and inclusion − that should inform design and planning decisions. These decisions have health effects at three scales: planetary, ecosystem and local. THRIVES is informed by theory and concepts from systems thinking (Meadows and Wright 2008), ecological health models (Rayner and Lang 2012) and ‘just sustainabilities’ (Agyeman 2013). It aims to inform research and practice in the fields of urban planning, architecture, urban design, engineering, transport, public health and others.

There are several key concepts communicated by the Framework, as articulated by Pineo (2020). First, the complex interconnections between urban environments and health can result in health impacts at multiple spatial and temporal scales that may not be immediately obvious to design teams and policy-makers. This complexity necessitates a systems thinking approach that recognises the counterintuitive and emergent behaviour in systems that are governed by feedback (Gatzweiler *et al*. 2018, Pineo *et al*. 2018b). Project teams need to be aware of the positive and negative impacts of design, planning and construction choices at all three scales of health impact (planetary, ecosystem and local) that are results of system interactions. For example, building design choices in a given city may affect the health of residents within and beyond the building; the health of ecosystems in and around the city, with attendant impacts on health; and of people and ecosystems around the world through processes such as anthropogenic climate change. As a result of this complexity, health and sustainability need to be considered in an integrated manner rather than as competing or separate objectives, resulting in silo-based design or assessment approaches. Integrated design can promote achievement of co-benefits across many objectives, whilst a narrow single-issue focus can create unintended consequences. For example, increasing the energy efficiency of housing must be accompanied by consideration of ventilation and cooling to avoid negative health impacts (Shrubsole *et al*. 2014). Second, the knowledge used to inform urban environment decisions should come from both scientific (and other technical) evidence and the situated knowledges of locally affected communities (Kumar 2002, Corburn 2005, Innes and Booher 2010, Agyeman 2013), described below. Finally, in recognition of the complexity of urban health systems, government, building managers and other responsible authorities should monitor urban environments (using participatory processes to define indicators) to ensure that policy and design intentions result in health-promoting places for all residents (Rydin *et al*. 2012).

The nature and form of knowledge production is being challenged to include a much broader set of voices to address complex societal problems (Nicolescu 2002, Stokols *et al*. 2013, Berger-González *et al*. 2016). Gibbons (1999) states that ‘reliable knowledge can only be socially robust if society sees the process of knowledge production as transparent and participative’ (p. C83). Over the last decade there has been increasing emphasis on participatory, collaborative and transdisciplinary processes within research, leading to new forms and types of knowledge production. Participatory methods include engaged research, citizen science, knowledge exchange, participatory action research, community-based research and public and patient involvement (PPI), with overlapping but distinct methods and focus areas. Funtowicz and Ravetz (1991, 2015) have advocated for incorporation of a broader set of views in the quality assurance of scientific processes from an ‘extended peer community’, which could be formed ‘not merely of persons with some form or other of institutional accreditation, but rather of all those with a desire to participate in the resolution of the issue’ (2015, p. 683). Different approaches have gained ground in topics related to the environment and health, notably citizen science, participatory action research, community based research and engaged research (Corburn 2005, Dennis *et al*. 2009, Israel *et al*. 2019).

Through a process of co-production, partners create more ‘relevant’ research questions, implementable outputs and potentially wide-reaching and significant impacts. Durose *et al*. (2018) argue that ‘Opening up science beyond scientists is essential, particularly where problems are complex, solutions are uncertain and values are salient’ − yet co-production is often under-valued and under-reported (p.32). By sharing different types of knowledge (i.e. disciplinary, lay and expert) it is possible to enhance all participants’ knowledge (i.e. mutual learning) (Wynne 1996). Wide collaboration can result in improved quality and social legitimacy of decisions and outcomes (Bailey *et al*. 1999, Holder 2004). Beyond research, the complexity of global health challenges, such as climate change and widening income inequalities, require the integration of multiple perspectives to formulate effective policy solutions (Corburn 2009, Innes and Booher 2010, Corburn and Cohen 2012, Buse *et al*. 2018). Public participation is advocated for health policy and service delivery (Martin 2009, Heritage and Dooris 2009) and urban development (Kumar 2002), with recognition that, in practice, such activities range from tokenistic (or manipulative) gestures to community-driven processes (Arnstein, [1969] 2019).

Calls for strong stakeholder engagement in urban governance are not new. Communicative planning theorists (Healey 1997, Sandercock 1998, Innes 2004) argued that: knowledge is value-laden, there is unequal distribution of power in planning and planners have a duty to represent the needs of disadvantaged communities. Innes and Booher’s (2010) DIAD (diversity, interdependence and authentic dialogue) theory of collaborative rationality highlights the importance of incorporating ‘lay knowledge’ in the development of policy solutions to complex problems which can result in ‘new knowledge and unanticipated policies and practices’ alongside systemic ‘changes in the values, goals, shared understandings, and the underlying attitudes of the participants’ (p.34). The need for public participation is echoed by healthy urban planning scholars. Collaboration with communities explicitly recognises that health inequities are caused by societal structures that are, in turn, influenced by built environment decisions which typically exclude those people who are most affected (Barton and Grant 2008, Corburn *et al*. 2014, Pineo *et al*. 2019). As a result, healthy urban design and planning processes should be inclusive of a wide range of knowledge sources to ensure that their outcomes promote health for everybody in society, not only those with the most agency and power.

Despite the potential benefits of research and policy co-production approaches, there are recognised challenges. In relation to research, transdisciplinary and co-production approaches have risks including: excessive time and cost, difficulty publishing, and potential threats to researchers’ psychological safety (Lynch 2006, Lang *et al*. 2012, Oliver *et al*. 2019, Black *et al*. 2019). In both research and policy contexts, there are diverse interpretations of suitable knowledge types to inform action, that can be driven by epistemological positions (Rydin 2007, De Leeuw *et al*. 2008). Among the professions and stakeholders involved in healthy urban development there are diverse perspectives about which knowledge types should inform decision-making. Fam and Sofoulis (2017) describe a case where engineers on a water and sewer infrastructure project to improve health in Alaska were reluctant to integrate community knowledge due to their preference for ‘positivist’, ‘hard’, and ‘black and white’ knowledge (p.1067). Pineo *et al*. (2020) recount the experience of an Australian public health practitioner who found legal adjudicators in planning disputes to be ‘hostile [toward] scientific method’ which resulted in decisions to allow developments that could harm health (p.9). These examples underscore the challenge highlighted by Carmichael *et al*. (2012, 2019), that diverse knowledge and conceptualisations of how the built environment impacts health are barriers to creating healthy places. In summary, identifying methods and frameworks for incorporating diverse forms of knowledge is important for research and policy-making related to healthy placemaking.

In presenting the THRIVES Framework, Pineo (2020) builds on previous assertions that healthy urbanism processes should be participatory, involving co-design and other methods to incorporate local knowledge. We have adopted the same thinking for the development of the Framework itself, including eliciting and responding to knowledge from built environment and health practitioners about the need for such a framework and the form that it should take. The initial drive to develop the Framework was significantly informed by a collaboration between the authors and Guy’s and St Thomas’ Charity (GSTC). GSTC are an urban health charity with a land and property portfolio that funds their charitable activities supporting the health of Londoners in Southwark and Lambeth. Part of their asset management strategy now involves improving health through this portfolio. We have adopted an action research and transdisciplinary approach to work with the Charity and its development partners to develop, test and implement the THRIVES Framework. This work in ongoing and this article focuses solely on the iterative development and testing of the Framework, which has been funded by the Charity.

This article outlines the processes of the production of the conceptual framework, which brought together a range of knowledge and expertise from professional communities, culminating in a participatory workshop to test and improve the Framework. We begin with an overview of the research approach. We then discuss informal feedback that influenced early iterations of the Framework. We devote the majority of the article to presenting and discussing results of a participatory workshop with built environment and public health practitioners, focusing on how the Framework should be articulated and implemented in practice. In conclusion, we discuss our reflections of participants’ views and the approach we have taken, including the implications for practice and future research. The conceptual basis for THRIVES is elaborated by Pineo (2020) in an article that has been published in tandem with this paper.

### Research approach

Our research approach, and the processes adopted to refine and test the Framework, are informed by action research and transdisciplinary approaches. Specifically, we have drawn upon the ‘extended peer review’ approach. According to Funtowicz and colleagues (Funtowicz and Ravetz 1991, 2015, Liberatore and Funtowicz 2003), extended peer review is the process of including a range of non-academic stakeholders with relevant expertise and experience in the processes of assessing and validating the quality of research. Liberatore and Funtowicz (2003) explain that: ‘A plurality of perspectives is considered as enhancing both procedural legitimacy (through inclusiveness) and quality of knowledge (through extended peer review)’ (p.149). Extended peer review also aims to ensure that the quality of research supports its application beyond academe, into policy or other uses (Funtowicz and Ravetz 2015). We have drawn upon the ‘extended peer review’ approach to test and improve the THRIVES Framework, as outlined below.

#### Scoping and development

The scoping and development of the THRIVES Framework involved several stages to elicit informal and formal feedback. The Framework was, and continues to be, shaped by practices on the ground. Scoping activities involved initial literature reviews and reflecting on a series of interviews. Experienced design and planning professionals (n = 30) were interviewed (and gave consent under an approved ethics process) between 27 May 2019 and 26 February 2020. Face-to-face and virtual interviews were conducted in China, England, USA, Australia, Sweden and the Netherlands. Hand-written notes from the interviews and results of the literature review were analysed to inform preliminary versions of the Framework, along-side reflections from HP’s experience as a practicing urban planner. Furthermore, interviews informed our interpretation of the preliminary workshop results as we considered how English practitioners discussed healthy built environment topics in comparison with international interview participants. A preliminary version of the Framework was presented at the Healthy City Design 2019 conference (Pineo 2019). Feedback received at this stage led to relatively minor changes, such as broadening the design and planning goal of noise pollution to acoustic comfort to reflect growing understanding of the potential positive impacts of soundscapes (Aletta *et al*. 2018). Finally, we organised a participatory workshop to bring together stakeholders, open up a dialogue and promote collaborative learning via extended peer review processes.

#### Participatory workshop with practitioners

Formal feedback was elicited in a half-day workshop on 29 January 2020 in London with built environment and public health professionals. Participants were purposively sampled and recruited through two routes. Individuals were invited if they had experience with integrating health and wellbeing into planning policy and/or new development (or regeneration) either through practice or research in built environment or public health fields. A selection of specialisms were also sought (e.g. transport, green infrastructure, inclusive design, air pollution, climate change, and others). The biographies of 400 Design Council Built Environment Experts (BEEs) were screened and 26 were invited. HP’s professional network was also screened using contacts on LinkedIn, Twitter and email and a further 31 people were invited (a total of 57 invitations were sent). Professionals outside of England were excluded due to financial constraints. Travel costs were reimbursed, lunch was provided and there was no other remuneration for participation. The workshop was approved through a departmental low-risk ethics process and all participants received an information sheet and consent form, and gave signed consent prior to participating. Participants also received a link to a video presentation and slides of the Framework in advance (Pineo 2019).

The aims of the workshop were to test the THRIVES Framework as follows: 1) to understand whether the Framework effectively communicated the concepts that the researchers intended, 2) to identify concepts that were missing or otherwise required adjustment, 3) to learn how the diagram could be improved and 4) to understand how the Framework could be used in practice or research. Alongside these aims, we also sought to share our new conceptual thinking with the audience and to influence their work with this knowledge.

#### Workshop participants

There were 26 professionals at the workshop, 20 of whom completed an optional demographic survey. Of those who completed the survey, 60% (12/20) described themselves as representing built environment professions, 35% (7/20) covered public health and one represented both (Table 1). Participants were able to select multiple options to any of the demographic survey questions. There was a spread across public and private sector organisations of different types; however, many participants selected more than one. The participants were primarily experienced professionals with 15 or more years in practice (70%, 14/20). There was a mixture of ages and genders represented, although of note, 65% (13/20) of participants who completed the form were aged over 50 (Table 2).

#### Workshop format

**Figure 2:**
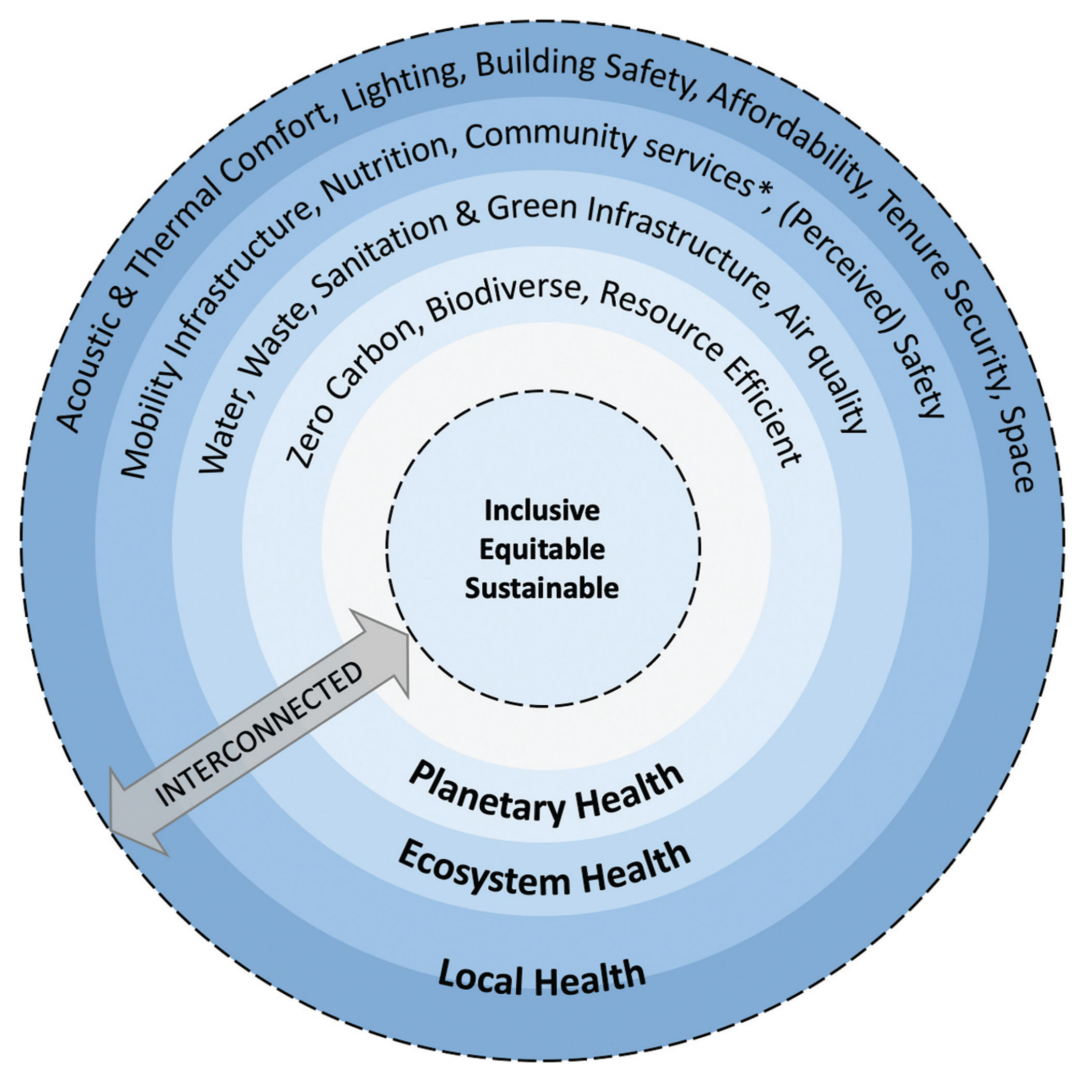
Preliminary healthy urban design and planning framework. *Community services denotes employment, education, cultural, retail, leisure, healthcare and other facilities.

The workshop was led by three experienced facilitators from the Design Council to introduce an element of independence from the researchers. We hoped that this would enable participants to feel comfortable in candidly describing their perspectives. We worked with the Design Council team to choose appropriate workshop activities to elicit participants’ views. The workshop agenda (Table 3) involved a networking lunch followed by a 15-minute presentation of the preliminary Framework (Figure 2). The preliminary version contained all capital letters and this was amended following workshop feedback for presentation in this article. The presentation covered the following points: the methods for developing the Framework, examples of other frameworks with emphasis on the Barton and Grant (2006) Health Map, and an explanation of the definitions and conceptual basis for the Framework’s three scales of health impact and core principles. Participants were seated in groups of four to six people, enabling partner and group activities.

**Figure 3:**
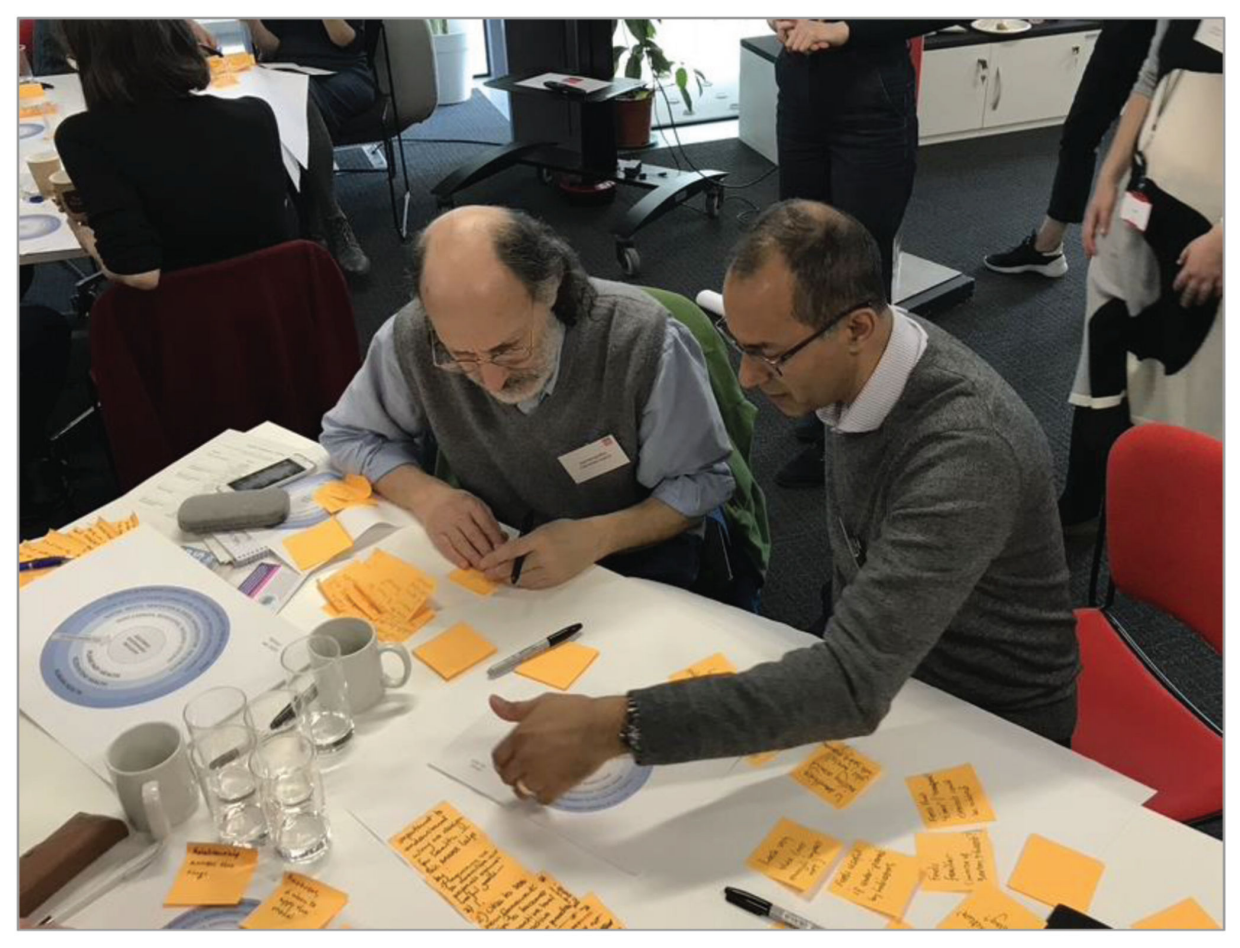
Participants doing the ‘Thinking-aloud’ exercise.

The first activity, called ‘Thinking-Aloud’ (Figure 3), involved participants working in pairs to either listen or speak, and then reversing roles. The listener wrote on post-it notes whilst the speaker described what they understood about the Framework. The speaker was asked to ‘simply verbalise their thoughts as they moved through the visual diagram’. The goal of this activity was to ‘discover what users of this Framework really think about the concepts it represents and the visual design’ with explicit recognition that the results would be used to ‘develop actionable redesign recommendations’. Following the paired activity, participants discussed their views with others among their table. Finally, they described their views to the group.

**Figure 4:**
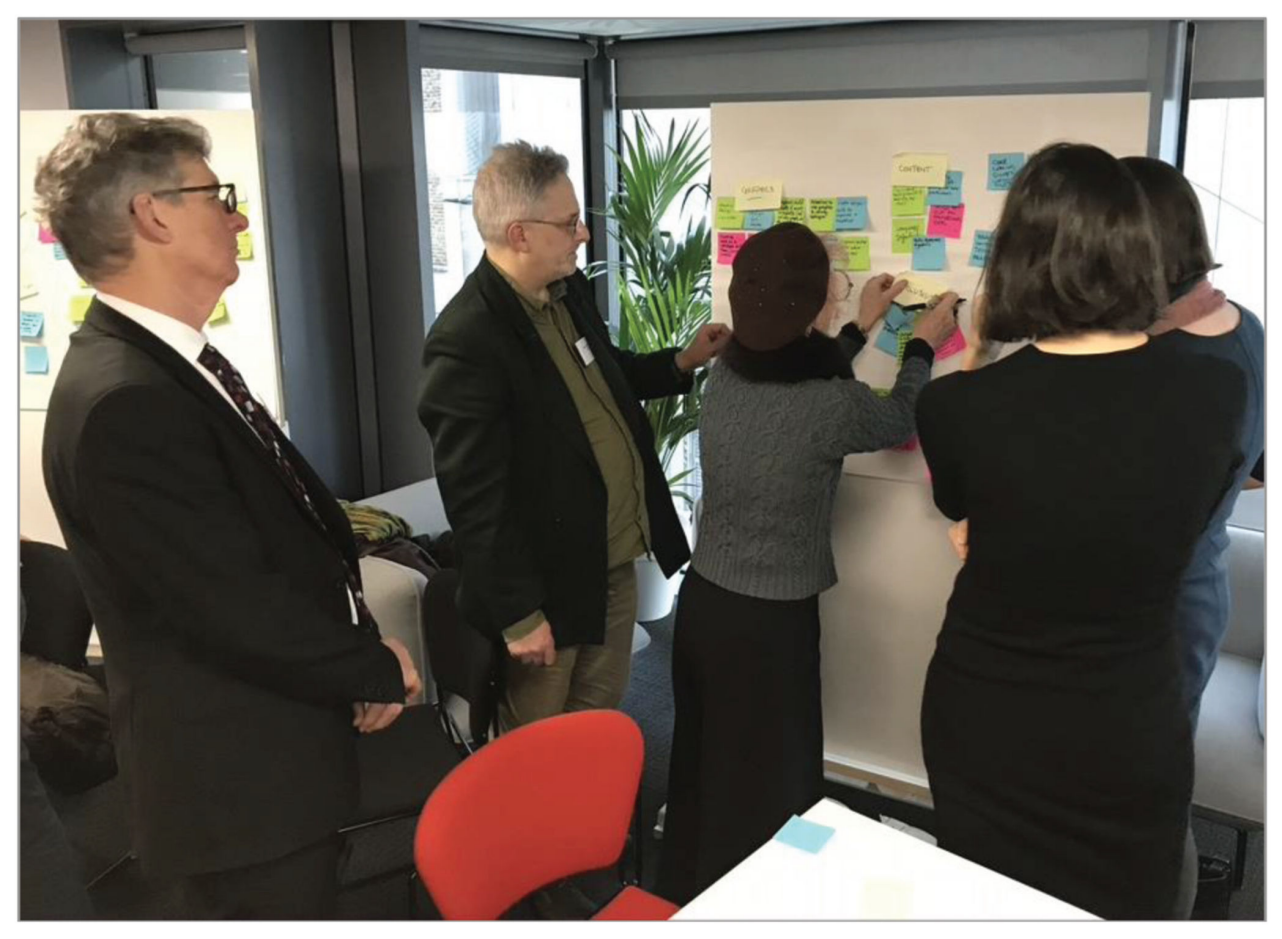
Participants finding themes in the ‘Rose, Thorn, Bud’ exercise.

The second activity, called ‘Rose, Thorn, Bud’ (Figure 4), involved participants using coloured post-it notes to describe what they liked (rose), what they did not like (thorn) and what could be improved (bud). Participants were given printed copies of Pineo’s presentation which included definitions of the three scales of health impact and the core principles. They worked independently to start with and then grouped their post-it notes on flip charts according to themes, which they chose. This resulted in clustered post-it notes of positive, negative and promising points (or those representing opportunities for improvement). Finally, groups shared their views with the room.

#### Data gathering and analysis

We gathered data throughout the workshop using researchers’ (GM and IB) hand-written notes using a template and participants post-it notes. The note taking template contained a number of prompts to focus the researchers’ attention (e.g. consider points of divergent thinking, consensus or confusion among participants). Researchers noted the following in a table: the part of the session being observed, discussions that ‘stand out’ (both positive and negative points), explanation of why the discussion ‘stands out’ (including any quotes or information that supports the observation). Following the workshop, GM collated participants’ feedback from post-it notes into a single document grouped by exercises and tables. GM and IB summarised their reflections from hand-written notes into a single document. We used a framework to analyse the data (i.e. participants comments and our reflections) that included overarching pre-defined themes used to sort and group the data into categories (i.e. positives, negatives, opportunities for development). However, this evolved once we were more familiar with the data (re-reading the notes) and additional sub-themes emerged. We met as a team to discuss our key reflections. Then, HP analysed this material looking for common themes related to design that were subsequently used to inform a brief for a professional graphic designer to improve the visual illustration of the Framework. In this paper, we report the summary feedback gathered from post-it notes and hand-written feedback that we have tabulated and grouped according to deductively and inductively derived themes.

### Participants’ views of the preliminary framework

Participants were broadly supportive of the conceptual message of the preliminary Framework and more critical of its visual design (the preliminary version) and its potential to influence built environment practitioners without further resources. There were contradictory statements among members of the group, and these are shown in the sub-sections below.

#### Visual portrayal of concepts

The group provided detailed views of the concepts communicated through the diagram, including how successful particular visual design components were at conveying information (Table 4). Some participants stated that the diagram felt familiar and this was portrayed as either good (‘*easy to look at*’) or bad (‘*detracts from [its] value*’) (Table 4, Familiarity). There was significant divergence in opinion about whether it is right to have core principles and planetary health at the centre, with participants both praising and disagreeing with this change from previous frameworks (Table 4, Central theme). Similarly, participants were divided about how well interconnections were conveyed through the diagram with some saying that there is ‘*recognition of multiple scales and their interaction*’ whilst others said that the diagram was ‘*missing the complexity*’ (Table 4, Interconnections and logic). The most widely agreed message was that the visual design needed improvement, for example through consideration of colour, avoiding capital letters, using pictorial information, and being clear on the use of dotted lines and colour gradation (Table 4, Design). Although many participants found that the diagram ‘*makes sense*’ and is ‘*intuitive*’.

#### Scales of health impact

The concept of three scales of health impact was praised by some participants, and perhaps disliked or not fully understood by others. Table 5 shows the\ divergent feedback and represents some points of misunderstanding. For example, one participant said ‘*some points in the ecosystem [scale] also have impact on human health*’. It was explained at the start of the workshop that the Framework intends to convey that all scales impact human health, yet this message was not fully understood. The order of scales was not agreed upon by all participants, as described above and in Table 5, where some felt that planetary health belonged at the outside of the ring.

#### Missing components or concepts

Participants identified areas for improvement and missing components of the Framework. There was some confusion about the meaning of missing design and planning goals and why they appeared at certain scales and not others. One participant wrote ‘*As a list of features* (…) *it is quite useful but some things missing (why?)*’. This participant also felt that stating a design and planning goal at one level was problematic if it could be influenced at multiple levels, observing ‘*so scales don’t work*’. Participants were prompted to note perceived missing items, and they noted the following social and economic determinants of health or outcomes: culture, poverty, diversity, accessibility, economic dynamics, community, life satisfaction, affordability, and sex (as opposed to gender). Perceived missing topics also included environmental factors: housing, transport, soil, health contamination, indoor air quality and blue infrastructure. In summary, one participant noted that it was ‘*unclear if elements are examples or are meant to cover all*.’

#### Recognition of the framework’s potential impact

The re-orientation of the Framework, with core principles and planetary health at the centre was seen as ‘*conceptually strong*’ and a ‘*paradigm shift*’ that would provide the ‘*ability to question wider structural issues around health*’. This was explained by the Framework’s potential impact in changing professionals’ thinking about how a development impacts health: it ‘*nudges towards thinking about the impact of development outside the boundary (i.e planetary health, inclusive, ethical procurement)*’ and it ‘*encourages people to look at issues holistically*.’ With regard to how the Framework could be further improved, one participant wrote that it ‘*could extend to include social value/thriving*’.

#### Applying the framework

As early as the first ten minutes of the group work, participants began discussing how the Framework could be used in practice: ‘*Lots of things coming together − I wonder how I would use it?*’ A key issue of concern was that the Framework did not provide enough information on its own, with participants suggesting accompanying indicators, tools and charts (Table 6, Tools). In contrast, some participants felt that the design and planning goals in the Framework were already a checklist and that this was negative: ‘*List form is a checklist − is this the sophistication of what we need to look at places?*’. However, other participants saw the Framework as having potential to achieve ‘*inter/trans disciplinary action and communication*’ and ‘*break down professional barriers*’. Furthermore, participants identified that it could inform local plans and health or environmental impact assessment (HIA/EIA) (Table 6).

### Reflections on participants’ views

Here we reflect upon participants’ views in the context of the evidence and theory that had informed the development of the Framework. We consider the meaning of the workshop results and how they informed changes to the visual diagram, alongside indications for its implementation.

#### Diverse professional lenses and languages

Participants approached the Framework with different lenses (e.g. public health, transport planning or architecture) and they felt these perspectives would influence how the Framework was understood, for example some would see immediate connections to the Barton and Grant (2006) Health Map, whilst others would be unfamiliar with the Framework terms. We believe that the discussion revealed some structures and norms that shaped the participants’ perspectives and we aim to challenge some of these perspectives, such as the inappropriate public policy focus on individual ‘lifestyles’ (Kelly and Russo 2018), as described in Pineo (2020). Many participants drew upon their experiences to consider how the Framework could be applied in different contexts. They raised the need for interdisciplinary collaboration to achieve a healthy urban environment and they identified the Framework as a tool to enable movement in this direction. We believe that the workshop discussions demonstrated that the THRIVES Framework is doing what we intended, however, the preliminary version required design adjustments. Table 7 summarises the changes we made to the Framework in response to participant feedback, in other words moving from Figure 2 (preliminary version) to Figure 1 (current version). We will continue to reflect on the importance of language and background knowledge in articulating the Framework (see Implementation).

#### Inversion of previous models

Although there were divergent opinions about the Framework’s central focus on core principles and planetary health, we decided not to change this component of the model after reflection and discussion. Pineo (2020) articulates the two reasons why this choice was taken: 1) global environmental degradation represents the greatest threat to our health at the global population level and 2) contemporary public health theory demonstrates the increased importance of our social and physical environment in determining our health. In correspondence with Marcus Grant and Hugh Barton, authors of the Settlement Health Map, they provide an explanation for the central and outer positions of people and global factors, respectively. ‘The Settlement Health Map stemmed originally from the simple models of sustainable development in the 1990s, with social at the centre and the environment, Earth ecology, around it, with economy acting, for good or ill, as the linking factor. Having the environment all round emphasises the ecological limits to growth. In developing this model by combining it with the social determinants of health, explicitly including the built environment, we wanted to keep the social dimension − people − at the centre. This has ensured that the model had, and continues to have, a wide resonance and use within the public health fraternity, who previously found it difficult to engage with built environment agendas. However, the Settlement Health Map remains a true eco-system model, and has shown its worth through practical use, in bringing together public health and built environment professions for common purpose’ (Barton H. and Grant M. pers. comm. 16 May 2020). We agree that this framing was appropriate in the early 2000s but a number of participants seemed to support the central focus of planetary health (and the core principles) which responds to shifting public and scientific concerns in recent years (see ‘Recognition of the Framework’s potential impact’).

#### Clarifying the role of the framework

Participants acknowledged, and considered a positive attribute, that the Framework shows the relations between a range of concepts and dimensions related to the built environment and health. The purpose of the Framework is not to portray very specific causal linkages among those dimensions, or to specific health outcomes. The Framework does provide a broad conceptual map of numerous interconnected attributes, which is why we view it as an integrative conceptual framework, not a design and planning checklist. A key role of the Framework is to encourage consideration about which attributes in the system are likely to have an impact at different scales − and the interactions between these attributes. However, we have sought to highlight these inter-connections to a greater degree in the current version of the Framework (Table 7).

Some participant agreed that the core principles of sustainability, equity and inclusion should be the basis for understanding and transforming the built environment to promote health. However, other participants saw this framing as subjective and open to interpretation or challenge from other economic or political positions. We do aim to challenge existing perspectives about how the built environment affects health and wellbeing, yet we recognise the contested nature of the concepts underpinning the Framework (e.g. equity). We will continue to work with practitioners to understand how they interpret and respond to these concepts.

#### Breadth vs Depth

The breadth of the Framework is a strength, yet it also creates difficulties in adequately describing and visually representing it concisely. The challenge of balancing complexity and oversimplification was expressed by participants. The Framework builds upon existing theories and practices, bringing points of reference to aid users in developing shared understanding and adapting the Framework to suit different locations and audiences. Although some practitioners focused on particular attributes or components, the discussion revealed that many practitioners are not looking for one solution, but to build multiple, linked strategies to improve health and wellbeing, acknowledging that the choice of strategies will depend on the starting point in any given context. In summary, in applying the Framework we hope that different audiences will collaboratively seek to understand: where we are (in a particular city or neighbourhood, or against a particular design objective), where we want to be (in terms of ideal design or planning outcomes), and how we can get there (specific strategies and related opportunities and challenges to implement these). In this sense, the Framework could move beyond being a tool for (re)conceptualisation, into being a catalyst for, and structure to support, active engagement. For example, it could support teams involved in new development or planning processes and their wider stakeholders with discussing, prioritising, and deciding how to plan and design healthy places.

#### Implementation

There was significant interest in how the Framework could be applied, which we perceive to indicate agreement with the underlying concepts. Participants were aware that implementation was a next phase of research. They identified the need for a set of example design strategies and indicators to support professionals with clarifying how a particular design or planning goal could be achieved in practice. The development of the THRIVES Frameworks was informed by a set of evidence-based indicators, as provided by (Pineo *et al*. 2018a, 2018b) for each of the design and planning goals and scales of health impact. We will consider how these indicators and the wider evidence base can be communicated effectively in conjunction with the Framework. Indicators would support use of the Framework in impact assessment (health, environmental or integrated), setting targets and monitoring progress. We will use the learning developed through application of the Health Map to inform our approach (Barton and Grant 2008, Grant and Barton 2013, Grant 2015).

## Discussion

This article has described our action research approach to developing and implementing the THRIVES Framework with particular focus on the results of a participatory workshop with built environment and public health practitioners. The workshop and wider research created opportunities to incorporate knowledge from a more diverse set of sources than may typically form conceptual framework development. It also provided an excellent opportunity for the researchers to share the latest thinking with the sector, and increase the relevance and acceptability of THRIVES Framework for practice. Based on workshop participants’ views, we are hopeful that the THRIVES Framework will help to overcome known barriers for healthy placemaking related to diverse knowledge and conceptualisations of how the built environment impacts health (Carmichael *et al*. 2012, 2019). In this section, we briefly discuss our positionality and the strengths and limitations of our approach. Then, we discuss the potential contributions of our research process for future participatory workshops (be they research- or practice-based). Finally, we consider how this research contributes to overcoming barriers to healthy urban development.

### Positionality of the researchers

Within action research processes, such as ‘extended peer review’, it is important to acknowledge the positionality, role and influence of the researcher to the research being undertaken (England 1994). Despite the workshop being facilitated by the Design Council, our roles, presence and positionalities (for example with respect to gender, race, class and regarding participants’ awareness of our involvement in the broader research project) are likely to have influenced the action research process − in terms of who participated, the views expressed, data collected and the knowledge produced. Nevertheless, through techniques including open questioning and encouraging dialogue within the workshop, we attempted to create conditions which would bring together and include different knowledges. Furthermore, we cannot ignore our perspectives, situated as we are within our urban planning, environmental geography and public health backgrounds and our experience has, of necessity, influenced our interpretation of the feedback collected. Our intersubjectivity will also be manifest in our decisions regarding changes to the Framework given that conflicting views were expressed by participants. In this paper, we have attempted to be transparent about our interpretations of the data and our assumptions.

### Strengths and weaknesses of the approach

We have identified several strengths and limitations to our approach for testing and developing the THRIVES Framework. As stated, we believe the participatory approach has supported the integration of multiple knowledge types. The independent facilitation by the Design Council team and the use of different discussion and elicitation techniques helped us gather significant data in a relatively short workshop. The Framework itself addresses an identified gap (Pineo 2020) and responds to needs expressed by research participants. Regarding limitations, we acknowledge that our purposive sampling on the basis of built environment and health knowledge may replicate existing biases in research and practice (for example, related to age, sex, class, race, disability). Our analysis is limited to the data that were either reported on post-it notes or observed by researchers. Finally, we were only able to involve professionals from England which limits the international perspective that the THRIVES Framework aims to address, particularly with regard to low-income settings. We reflect further on the process and outcomes of ‘extended peer review’, and the selection of this method below.

### Contributions for participatory workshops in research and practice

Reflecting on the participatory *process* that we adopted, we feel that there are a number of lessons that may be of use to researchers or practitioners carrying out such workshops. First, we noted that even in the context of a relatively contained ‘extended peer review’ process it was important to be transparent about the purpose, scope and boundaries of the process. This builds on key principles of public participation and helps build trust among participants and workshop conveners. Second, we felt that having varied group sizes for different exercises supported multiple objectives. Participatory and collaborative practices tend to require shifts in ways of working for people, professions and organisations. Common barriers to participation and collaboration for healthy planning include: time, capacity, resources, language and difficulty identifying shared goals (Carmichael *et al*. 2012, 2019, Design Council 2018, Pineo *et al*. 2020). Activities in our workshop ranged from exercises in pairs and small groups (5−6 individuals) and whole-room feedback (as detailed in Table 3). We believe that this variation helped to: build trust between participants; break down professional/disciplinary barriers and (potential) perceived power differentials; promoting active, critical engagement with the Framework; and enable all participants to contribute, including more introverted individuals. A third lesson is that despite these benefits, there were potentially some challenges related to inter-personal dynamics and ability for all members to share their knowledge within the group. In future, we would move participants to different groups between the two main exercises to ensure that different voices were heard within and beyond each group.

Considering the *outcome* of ‘extended peer review’, we believe that this approach has resulted in a significantly different visual representation for the THRIVES Framework than we would have otherwise produced. We greatly appreciated participants’ specific ideas to shape and communicate the THRIVES Framework. Participants were constructively critical and we perceived their feedback to be genuine. Within this participatory approach, it is important to mention we intended for this process to impact participants’ knowledge (and potentially their practice) beyond the development of the Framework. We did not ask participants to reflect on the workshop process itself, therefore we cannot report how it may have shifted their perspectives. In retrospect, we felt that doing so may have provided additional useful insights for participants and us, as researchers. A fourth lesson to share to future workshop conveners is to build in time for participants to reflect on the process and their learning.

There are many different participatory methods and tools that are effective in generating different outcomes for action research or healthy urban development processes, not all of which would have been effective for our purposes. For example, the Design Council (2015) Double Diamond framework is valuable for idea generation; the Delphi Method and Nonimal Group Technique can be useful for prioritisation and decision-making (Hsu and Sandford 2007, Foth *et al*. 2016); and citizens’ juries can support consensus building (Street *et al*. 2014). Other approaches are more suited to longer-term collaboration between urban stakeholders, such as co-operative inquiry (Heron and Reason 2008) and experimentation-oriented and transdisciplinary approaches such as urban living labs and CityLabs (Kronsell and Mukhtar-Landgren 2018, Culwick *et al*. 2019), which help participants explore new viewpoints, ways of working and develop shared understandings within a particular urban context. Finally, game- and simulation-based approaches such as the SUSTAIN Game-Based Learning on Urban Sustainability project and Climate Interactive’s EnROADS policy simulation model offer innovative ways to catalyse critical and interactive learning and knowledge production (SUSTAIN 2020, Climate Interactive 2020). In selecting ‘extended peer review’ we sought to match our research goals with practical boundaries, such as participants’ time and ability to collaborate. We are satisfied that this approach fulfilled our goals for testing the THRIVES Framework. Even though participants were experts in healthy urban environments, we are hopeful that they broadened their perspectives and/or professional network as a result of the workshop.

### Overcoming barriers to healthy placemaking

In relation to designing and planning healthy cities, we believe that the process and outcomes of this research can help to overcome existing barriers. A key barrier is lack of shared understanding among professionals about how health relates to the built environment (Carmichael *et al*. 2012, 2019). This links to a further barrier that professionals lack knowledge about how places affect health for different groups, such as racial minorities or women (Loukaitou-Sideris and Fink 2008, Lusk *et al*. 2019). The THRIVES Framework was developed by incorporating diverse forms of knowledge and we believe it can be used in practice to build shared understanding about healthy placemaking. THRIVES provides a structure for understanding and (re-)conceptualising healthy urban environments. Policy-makers or design team professionals may use the Framework for discussion and debate about what healthy design and development means for a particular project or policy. Likewise, the Framework could be used by (or with) community representatives in participatory design and planning process. Stakeholders are likely to have different perspectives and background knowledge about how the built environment impacts health, and these can be drawn out through discussion using the methods that we have discussed in this paper. The Framework may also support education and research activities that can build shared understanding. Building upon the enthusiasm of workshop participants and the Guy’s and St Thomas’ Charity, the authors (GM and HP) are in the early stages of co-creating a training programme with and for professionals who seek further knowledge about integrating health and wellbeing into urban developments, specifically through the THRIVES Framework.

There are wider barriers to healthy urbanism that require examination, specifically the perception of increased costs for development and operation. This perception is discussed widely in academic (Carmichael *et al*. 2012, Pineo *et al*. 2020) and practitioner literature (Chang 2018, Pineo and Rydin 2018) and occurs across markets and types of development (e.g. residential, office, etc.). The costs and benefits of achieving healthy development are distributed across a wide range of actors making it difficult to easily demonstrate the ‘business case’ (Pineo and Rydin 2018). In many countries, this issue results from the reliance on private sector developers to deliver healthy places within the margins that can be reasonably expected from such investments (Rydin 2013). A range of government interventions can shift the current dependency on private actors to voluntarily create healthy and sustainable environments including increased regulation, public-private development partnerships and financial incentives for developers. Where these mechanisms are not applied, practitioners and researchers who are interested in progressing healthy and sustainable development need to demonstrate that there are no-or-low cost design solutions that can be adopted at all development scales and types, and these are usually best integrated at the earliest stages of planning and design. We hope that the THRIVES Framework can aid these conversations by broadening practitioners’ understanding of which design measures will support health and by demonstrating the need to think of health impacts beyond the boundaries of new development. We recognise that the Framework could be criticised for promoting an unachievable utopian vision, or downplaying potential tensions between goals and principles (Been *et al*. 2010). Nevertheless, the challenges that motivated the development of this Framework are real and urgent and (what some might see as radical) solutions are required.

## Conclusion

In conclusion, the participatory extended peer review approach taken in this project made it possible to gather and incorporate practitioner knowledge into the development of the THRIVES Framework, as well as acting as a sounding board to help maximise the Framework’s relevance and utility for practitioners. The approach outlined in this paper relates to wider calls for bridging diverse knowledges and co-producing new knowledge between researchers, professional communities and citizens to create health-promoting built environments. Our example shows how active participation and co-production can happen, albeit in a relatively contained exercise compared to the long-term processes of urban development. Our reflections have implications for effective engagement in the field of healthy urbanism: diversifying knowledge in the research process; creating platforms for participation; forming networks of practitioners; and building collective knowledge. Based on our findings during this process, we believe that the Framework offers a way to bridge the divides − be they conceptual or disciplinary − faced by built environment and public health professionals alike, and to reconceptualise what healthy place making means in the 21^st^ century. Such a paradigm shift will be essential if we are to solve the most urgent environmental and health challenges we face and transform our towns and cities into vibrant, inclusive places that sustain human and planetary health alike.

## Figures and Tables

**Table 1:** Count of professions represented at the workshop

Profession and employment	Count of options represented*		Count of participants by option	
**Profession(s)**	**Sub-total**	**Total**	**No.**	**%**
**Built Environment**	-	19	12	60%
Planning	8		-	-
Architecture	3		-	-
Urban design	3		-	-
Other (not specified)	3		-	-
Landscape architect	1		-	-
Access consultant	1		-	-
Public health	8	8	7	35%
Built environment and public health	-	-	1	5%
**Employment organisation(s)**				
**Public sector**	-	10	-	-
Local government	5		-	-
National government	2		-	-
Other public sector	3		-	-
Private sector	-	5	-	-
University	-	4	-	-
Self-employed	-	4	-	-
Charity	-	1	-	-
Unemployed	-	1	-	-

Note that the count of professions represented is the number of times each profession/employment organisation was selected by participants. Participants were able to select multiple answers. No.: Number.

**Table 2:** Participant demographics.

Characteristic		Count/proportion of participants by option	
		No.	%
Years of experience in the profession	Less than 5 years	2	10%
	5 to 10 years	3	15%
	10–15 years	1	5%
	More than 15 years	14	70%
Age	20–30	2	10%
	31–40	2	10%
	41–50	3	15%
	51–60	7	35%
	Over 60	6	30%
Gender	Female	9	45%
	Male	11	55%

Other options included ‘other’ and ‘prefer not to say’ and these were not chosen.

**Table 3:** Participatory workshop agenda.

Time	Activity	Lead
13:30	Arrival, Lunch and Networking	N/A
14.00	Welcome and Introductions	DC staff
14.10	Overview − Healthy Urban Design and Planning Framework	Helen Pineo
14.20	Q&A	
14:30	Group exercise 1: ‘Thinking-aloud’ Testing Working in pairs (20 mins), Working in groups (20 mins), Giving summary of feedback to the room (20 mins)	DC staff
15.30	Break	N/A
15:40	Group Exercise 2: ‘Rose, Thorn, Bud’ Working individually (10 mins), Working in groups to cluster & theme (20 mins), Giving summary of feedback to the room (15 mins)	DC staff
16:25	Workshop summary & Next steps	Helen Pineo
16:30	Close	DC staff

DC: Design Council.

**Table 4:** Representative examples of critical and positive design-related feedback received on post-it notes.

Theme	Examples of critical feedback	Examples of positive feedback
Familiarity	‘*Looks very similar to others [which] detracts from [its] value*’ ‘*How will it help me? − looks obvious; − not stimulating*’	‘*Looks like familiar model so easy to look at*’ ‘*Looks like existing frameworks, not new, familiar to public health*’
Central theme	‘*Inside out? Planetary health should be the biggest rather than the core*’ ‘*The order should be human health in the middle*’ ‘*Association with other models makes the eyes want it to be the inverse*’	‘*Equitable, sustainable and inclusive are good core principles*’ ‘*Planetary health at core [is positive]*’ ‘*Good to have equitable at the centre to compared to traditional model i.e. individual at the centre*’
Inter-connections and logic	‘*Would a mind map be more helpful − arrows?*’ ‘*Develop logic and coherence – hierarchy, arrows*’ ‘*Difficult to understand the hierarchies*’ ‘*Missing the complexity* ‘*Restrictive*’	‘*Recognition of multiple scales and their interaction*’ ‘*Demonstrates inter linkages in a holistic way, whole system way)* ‘*Looks like a lot of info at first but clear when you go through if* ‘*Feels like a good high-level framework*’ ‘*Complex, captures many relevant issues and make sense, intuitively*’
Design	‘*Do not use block caps*’ ‘*Blue too [National Health Service]*’ ‘*Visual is difficult for non-health community*’ ‘*Not pictorial therefore not immediate engagement without instructions*’ ‘*Mixture of graphical information (dotted lines and arrows, gradation of colour) what is the hierarchy of information*’	‘*Good colour coding*’ ‘*Circular is good*’ ‘*Multiple layers is good − planetary to human*’ ‘*It makes sense*’

**Table 5:** Representative examples of critical and positive feedback about scales of health impact received via post-it notes.

Critical feedback	Positive feedback
‘*Some points in the eco system also have impact on human health*’	‘*Embracing all scales, and encouraging us to consider global to local*’
‘*Planetary health should be the outer ring − the order is arbitrary*	‘*It makes comprehensive connections across scales*’
‘*Don’t understand first two levels of human health*’	‘*Brings together human, eco, planetary health and tries to link it to levels of action and influence*’

**Table 6:** Representative examples of critical and positive feedback about implementing the Framework grouped by themes.

Theme	Critical feedback	Positive feedback
Immediate route to application	‘*Does not generate solutions*’ ‘*Doesn’t suggest avenues to explore i.e. actions*’ ‘*Factors related to decision making could be added*’ ‘*Needs more consideration of politics and politicians (N)*’ ‘*Everywhere is different*’	‘*Could help or inform future local plans*’ ‘*Scale is relevant to practice. Health connects people together*’ ‘*Could really support population and human health chapter in EIA*’ ‘*Connecting this to value and benefits makes it practical*’ *’Useful framework for HIA*’
Tools	‘*What are the indicators? How to measure outcomes?*’ ‘*Needs to connect to tools and measures*’ ‘*Needs a chart that connects it with reality*’	‘*Could be a great tool for achieving inter/trans disciplinary action and communication, focused on an issue*’ ‘*This process helps by: allowing us to describe our proposals against useful goals*’
Stakeholder collaboration	‘*Needs buy in from multiple organisations, challenging*’ ‘*Language important for shared understanding*’ ‘*Silo working how to identify and capture right groups to engage*’	‘*The integrated approach to health has potential to break down professional barriers*’ ‘*Has the potential to be used by a wide range of actors in built environment*’ ‘*Could connect different disciplines*’

**Table 7:** Paraphrased participant feedback about the Framework design and summary of changes between preliminary and current versions.

Paraphrased participant feedback	Summary of changes to the preliminary Framework
Overall design needs improvement (colours, interconnections across scales and design/planning goals, avoiding capital letters, meaning of lines).	Procured professional graphic designer to support improvements to the visual diagram.
Needs to differentiate scales of decision-making from health impact scales.	Inserted new scales that are associated with urban built environment decision-making.
Lack of clarity about comprehensiveness of design and planning goals and association with each scale of health impact.	Used design and narrative description to indicate that design and planning goals are examples at each scale.
Inconsistency in design and planning goals with inclusion of outcomes (physical activity) and built environment components (water infrastructure).	Changed words to create consistency across terms.
Opportunity to clarify the purpose of the Framework and integrate wider sense of social value and thriving	Changed name from ‘healthy urban design and planning framework’ to THRIVES − Towards Healthy uRbanism: InclusiVe Equitable Sustainable.

## Data Availability

The data that support the findings of this study are available on request from the corresponding author, HP.
